# A visible-light photodetector based on heterojunctions between CuO nanoparticles and ZnO nanorods

**DOI:** 10.3762/bjnano.14.84

**Published:** 2023-10-13

**Authors:** Doan Nhat Giang, Nhat Minh Nguyen, Duc Anh Ngo, Thanh Trang Tran, Le Thai Duy, Cong Khanh Tran, Thi Thanh Van Tran, Phan Phuong Ha La, Vinh Quang Dang

**Affiliations:** 1 Faculty of Materials Science and Technology, University of Science, Ho Chi Minh City 70000, Vietnam; 2 Vietnam National University (VNU-HCM), Ho Chi Minh City 70000, Vietnam; 3 Center for Innovative Materials and Architectures (INOMAR), Ho Chi Minh City 70000, Vietnam

**Keywords:** CuO nanoparticles, heterojunction, optoelectronics, visible-light photodetector, ZnO nanorods

## Abstract

Optoelectronic devices have various applications in medical equipment, sensors, and communication systems. Photodetectors, which convert light into electrical signals, have gained much attention from many research teams. This study describes a low-cost photodetector based on CuO nanoparticles and ZnO nanorods operating in a wide range of light wavelengths (395, 464, 532, and 640 nm). Particularly, under 395 nm excitation, the heterostructure device exhibits high responsivity, photoconductive gain, detectivity, and sensitivity with maximum values of 1.38 A·W^−1^, 4.33, 2.58 × 10^11^ Jones, and 1934.5% at a bias of 2 V, respectively. The sensing mechanism of the p–n heterojunction of CuO/ZnO is also explored. Overall, this study indicates that the heterostructure of CuO nanoparticles and ZnO nanorods obtained via a simple and cost-effective synthesis process has great potential for optoelectronic applications.

## Introduction

Optoelectronics is a field to accelerate the development of many technologies in the future, such as solar cells [1–2], light-emitting diodes (LEDs) [3–4], laser diodes [5], and optical fibers [6]. Optoelectronics devices contribute to meeting requirements in telecommunications, medical equipment, sensors, and military services. Among those applications, photodetection is an attractive area because photodetectors are the critial component to convert photon energy into electrical signals based on a nonlinear interaction between electromagnetic field and material surface [7]. Currently, many scientists are studying this topic in order to apply photodetectors (PDs) to “Industry 4.0”, which may include image sensors, biomedical imaging, manufacturing process control, environmental sensing, and optical sensors [8]. Various materials for photodetectors have been developed. Photodetectors can be classified into two main categories, namely PDs that work at a particular wavelength [9–14] and broadband PDs that work over a wide wavelength range [15]. Many researchers have focused on developing sensing materials operating in the visible-light region because this region is the biggest fraction of the solar spectrum (around 43%) [16–18]. Semiconductors are the heart of photodetectors as their bandgap allows for the absorption of photons in the desired wavelength range [19]. There are many semiconductor materials developed for this application. Among them, zinc oxide (ZnO) has been studied extensively over the last decades because of its large exciton binding energy of 60 meV at room temperature [20], excellent chemical and thermal stability, high electron mobility, non-toxicity, low cost, and simple synthesis [21–22]. Various shapes of ZnO nanomaterials can be easily obtained by controlling synthesis conditions (e.g., temperature, concentrations of chemicals, and annealing time). ZnO nanorods and nanowires have attracted great interest in photodetectors because their chemical and physical properties are exceptional for electronics applications, and their fabrication strategies are more facile than those of other structures [21]. Regardless, pure ZnO still has the considerable drawback of a wide bandgap (ca. 3.35 eV), which limits its usability for visible-light photodetectors. ZnO absorbs light only in the UV region (less than 4% of the sunlight spectrum) [23]. Extending the operation range of ZnO nanomaterials toward the visible range is still a challenge regarding the widespread use of this nanomaterial.

Traditional methods to modify ZnO, such as doping with transition metals [24] and decorating with noble metals [25], offer additional flexibility. Doping can significantly influence the optical and electrical properties of ZnO nanostructures, such as bandgap or conductivity [26]. Decorating ZnO with metals such as Ag, Au, Pd, Pt, and Al [27–28] can provide surface plasmonic effects that assist the electron transfer process in materials and extend the light absorption range of a photodetector [29–30]. However, these methods still face problems, including the requirements of controlling defects, scale-up for mass production, or troubles relating to decoration uniformity [31–32]. Another method is to form heterojunctions of ZnO and other narrow-bandgap semiconductors (NiO [33], PbS [34], CdS [35], and MoS_2_[36]) to extend the light absorption towards the visible region. Copper oxide (CuO) is a candidate because of its narrow bandgap (ca. 1.35 eV), which is suitable for visible-light detection. Hence, CuO can be a potential material for solving the problem of limited light absorption of ZnO. Conduction and valence bands of CuO are at a more negative potential than those of ZnO to form type-II region bonds between CuO and ZnO, avoiding recombination and accelerating the separation of photogenerated electron–hole pairs [37].

To explore and confirm the effects of combining CuO and ZnO, we developed a photodetector based on CuO nanoparticles (CuO NPs) and ZnO nanorods (ZnO NRs). CuO NPs were loaded onto ZnO NRs by a cost-effective, simple hydrothermal method at low synthesis temperature [38]. The CuO/ZnO photodetector was characterized, and its sensitivity was evaluated regarding visible-light wavelengths, including 395 nm (purple), 465 nm (blue), 532 nm (green), and 640 nm (red). Our device exhibited a high photocurrent of 10.4 μA and good responsivity (1.38 A·W^−1^) at 2 V bias. Although this is a fundamental study, it highlights the potential of the CuO/ZnO heterostructure for visible-light photodetectors and paves the way for developing more stable and effective visible-light photodetectors with remarkable photoresponse and shorter rise and decay times.

## Results and Discussion

**Figure 1:**
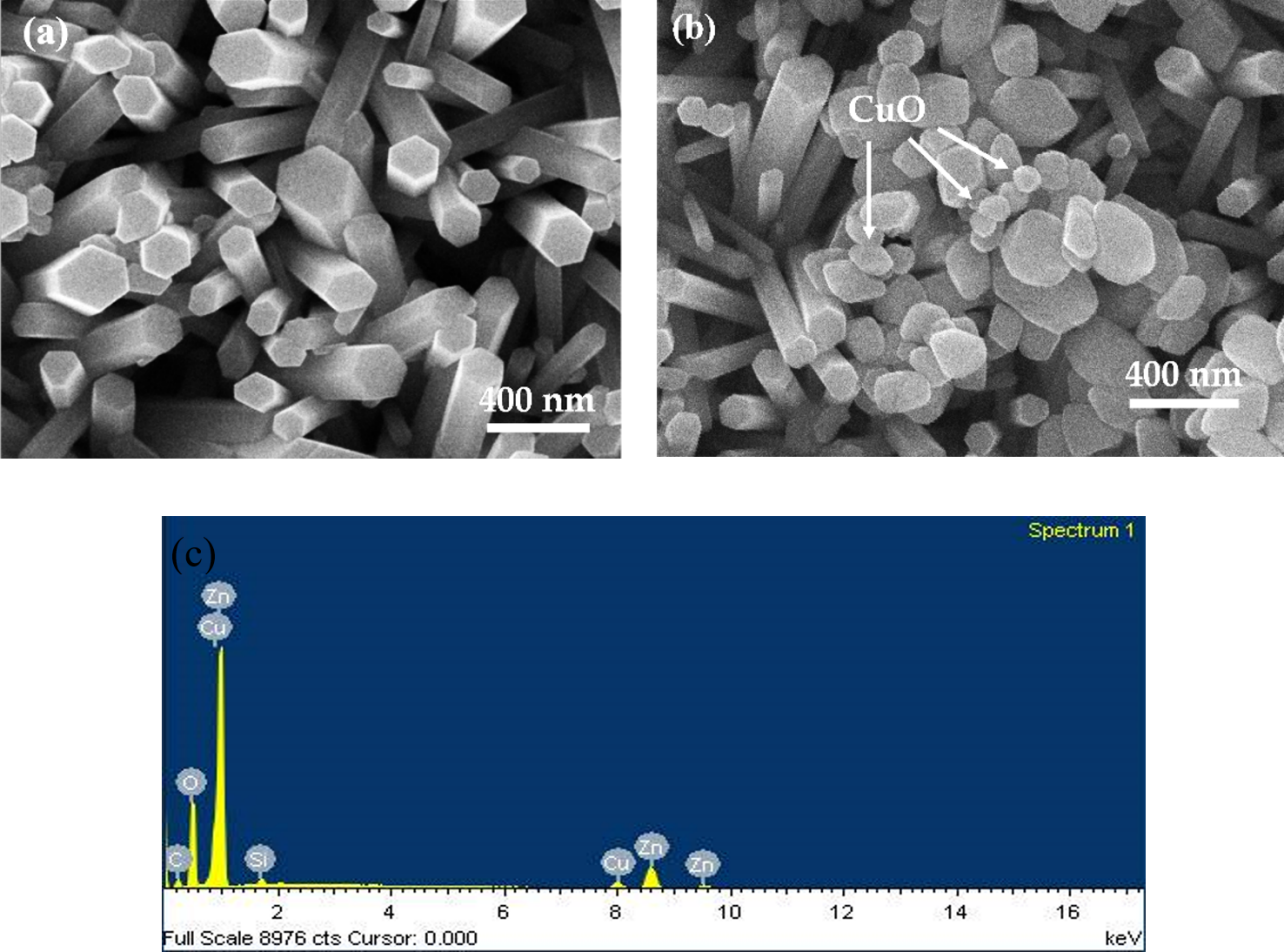
FESEM images of (a) pure ZnO NRs and (b) CuO NPs/ZnO NRs; (c) EDS spectrum of CuO NPs/ ZnO NRs.

The morphologies of pure ZnO NRs and CuO NPs/ZnO NRs were examined via scanning electron microscopy (Figure 1). The field-emission scanning electron microscopy (FESEM) image of ZnO NRs exhibits nanorods with hexagonal cross section, well aligned with the glass substrate (Figure 1a). Figure 1b indicates that many spherical nanoparticles are formed on the ZnO NRs after spraying the CuO NP solution with a concentration of 0.05 M. The energy-dispersive X-ray spectroscopy (EDS) results in Figure 1c show Zn, Cu, and O, which indicates the presence of ZnO and CuO. No further impurities were found.

**Figure 2:**
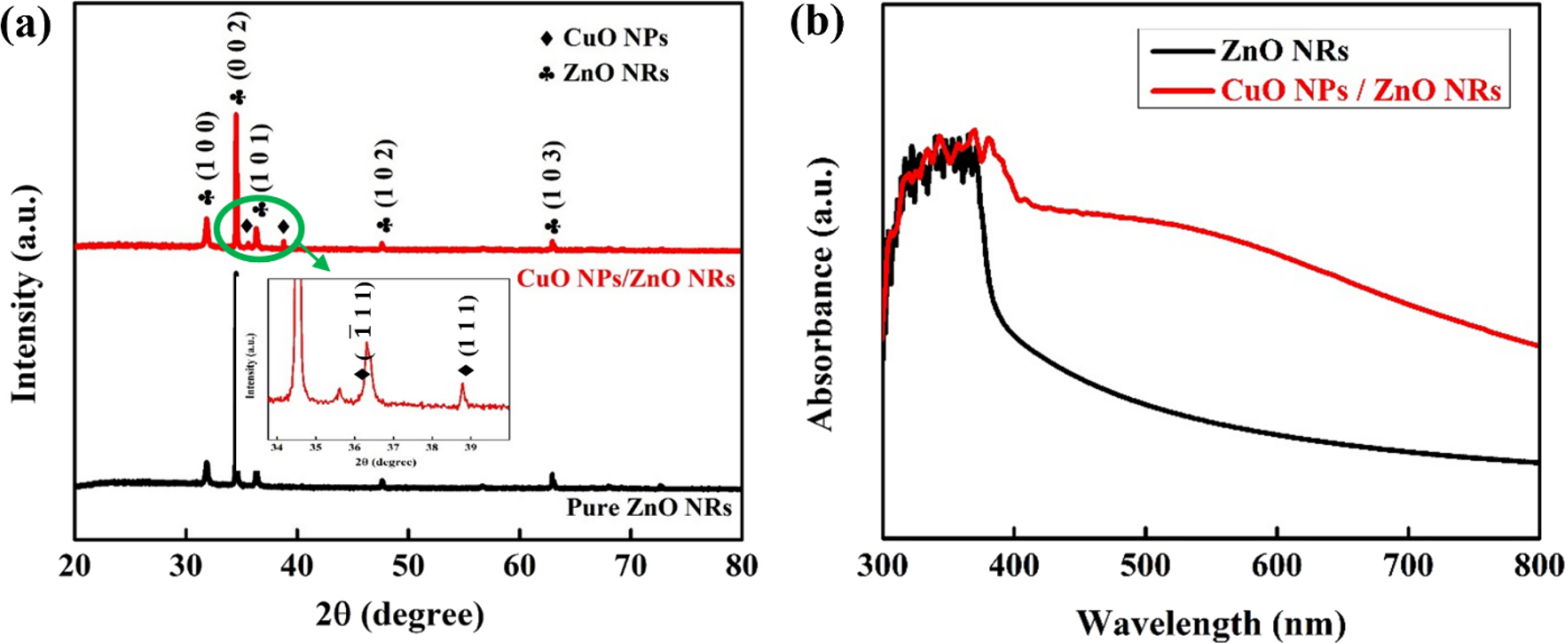
(a) XRD patterns and (b) UV–vis absorption spectra of the pure ZnO NRs and CuO NPs/ZnO NRs.

The results from X-ray diffractometry (XRD) and UV–vis absorption spectroscopy confirm that the nanoparticles covering the surfaces and edges of ZnO NRs are CuO NPs. Figure 2a shows XRD patterns of pure ZnO NRs (black line) and CuO NPs sprayed over ZnO NRs (red line) samples. The black line shows diffraction peaks at 2θ = 31.85°, 34.51°, 36.31°, 47.61°, and 62.89°, which correspond to the (100), (002), (101), (102), and (103) planes of hexagonal ZnO, similar to data from JCPDS-36-1451 and results reported before [39–40]. Additional peaks at 2θ = 35.90° and 39.14° appear in the XRD pattern of CuO NPs/ZnO NRs, while the original peaks of ZnO remain unchanged. These peaks were assigned to the (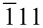) and (111) planes of CuO, consistent with JCPDS card No. 01-080-0076. This is proof of the existence of CuO NPs in the CuO NPs/ZnO NRs structure. Figure 2b shows the UV–vis absorption spectra of the pure ZnO NRs and ZnO NRs decorated with CuO NPs. Both samples containing ZnO NRs have absorption edges in a wavelength range from 300 to 380 nm due to the bandgap of ZnO (*E*_g_ = 3.2 eV). Especially, the CuO NPs/ZnO NRs sample exhibits absorption in the visible-light region. Decorating CuO NPs on ZnO NRs allows for more light scattering and absorption because the material’s surface and edges become rougher than the flat surface of pristine ZnO NRs [41].

**Figure 3:**
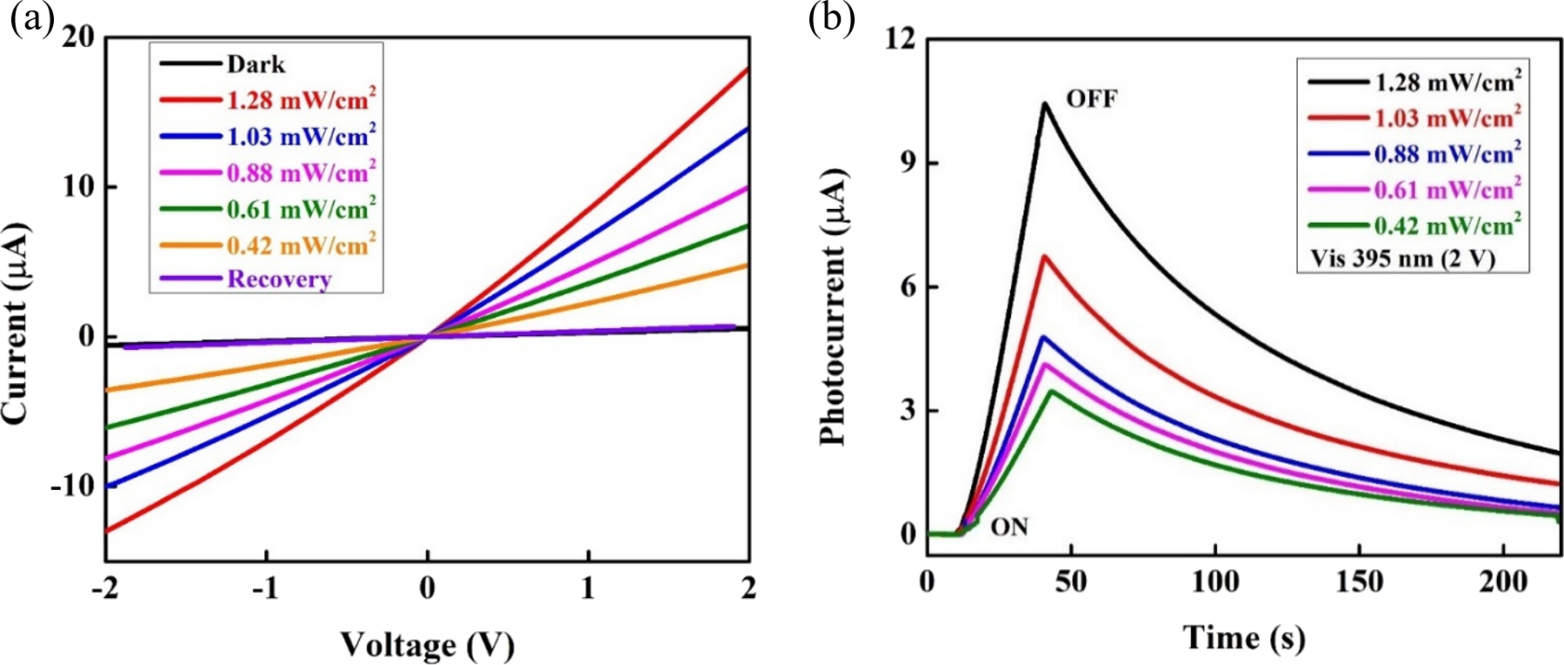
(a) *I*–*V* characteristics and (b) *I*–*t* curves of the photodetector based on CuO NPs/ZnO NRs heterojunctions under 395 nm exposure with different light intensities.

The optical characteristics of the device were examined through *I*–*V* and *I*–*t* measurements. Figure 3a shows *I*–*V* characteristics of a fabricated photodetector based on CuO NPs/ZnO NRs under visible-light exposure (395 nm) with different intensities. The device exhibits symmetrical and linear *I*–*V* relations under reverse and bias voltages, indicating a good ohmic contact between semiconductor materials and Ag electrodes [42]. It is worth noting that the current rise corresponds to the light intensity increase. The highest photocurrent reached 18 μA under a light illumination of 1.28 mW·cm^−2^. The low dark current can be explained by the formation of a depletion region near the surface of the ZnO NRs resulting from oxygen absorption [43] and at p-type CuO NPs/n-type ZnO NRs heterojunctions under dark conditions. In addition, the recovery current obtained 1 min after turning off the visible light is 0.7 μA, approximately the dark current, which shows the excellent recovery ability of the device.

The time-dependent photoresponse at a constant bias of 2 V under 30 s illumination with 395 nm light at different intensities is presented in Figure 3b. When the light is turned on, the photocurrent through the device considerably rises. The value of the photocurrent rises with the increase of the light intensity according to the formula [44] *I*_ph_ = *AP*^θ^, where *I*_ph_ is the photocurrent, *A* is a constant wavelength, *P* is the light intensity, and θ is a constant related to the photosensitivity of the device. The photocurrent can be calculated by the formula [17] *I*_ph_ = *I*_light_ – *I*_dark_, in which *I*_light_ is the current observed under visible-light illumination, and *I*_dark_ is the current under dark conditions. The calculated photocurrents under 395 nm illumination are 10.4, 6.73, 4.78, 4.11, and 3.47 μA at 1.28, 1.03, 0.88, 0.61, and 0.42 mW·cm^−2^, respectively, owing to the fast charge separation at the p-type/n-type heterojunctions of CuO/ZnO. The separation of electron–hole pairs prolongs the lifetime of free electrons, leading to an enhanced photocurrent [45]. When the applied voltage is fixed at 2 V, increasing the visible-light intensity promotes more excited electrons in the conduction band (CB) of CuO NPs. These electrons transfer to the CB of ZnO NRs and increase the photocurrent collected by the Ag electrodes.

Response time and recovery are essential when evaluating a photodetector’s performance. The response time is defined as the time to approach 63% of the maximum recorded photocurrent, while the recovery time is the time to decay to 37% of the highest value of the photodetector [46]. Under the 395 nm light illumination, response time and recovery time are estimated at about 21.38 s and 84.64 s, respectively. The fall time is relatively long because the photodetector is influenced by the persistent photoconductivity (PCC) effect [42]. The origin of PCC are adsorption and desorption processes of oxygen molecules. The re-adsorption rate of oxygen molecules is slow and a stable state is difficult to reach, which increases the required decay time [47].

To evaluate the photodetector performance, some essential parameters are considered. The responsivity (*R*) is used to determine the applicability of the visible-light photodetector. *R*, which is defined as the photocurrent divided by the product of power and area, can be calculated by using Equation 1 [48]:


[1] $$R=\frac{I_{\text{ph}}}{AP}.$$

The photoconductive gain (*G*) is the other important parameter of a photodetector; it can be determined via Equation 2 [49]:


[2] $$G=\frac{hc}{e\lambda }R.$$

The detectivity (*D*) describes the ability of a photodetector to detect weak optical signals. *D* can be calculated by Equation 3 [50]:


[3] $$D=\frac{R}{\sqrt{\frac{2eI_{\text{dark}}}{A}}}.$$

Here, *I*_ph_ is the observed photocurrent, *I*_dark_ represents the current observed under dark conditions, *e* is the electron charge, *P* is the illumination power density, and *A* is the channel area of the device (6 × 10^−3^ cm^2^). When the light density changes from 0.42 to 1.28 mW·cm^−2^, the highest *R* value is 1.38 A·W^−1^ at 0.42 mW·cm^−2^. At the same time, the measured values of *G* and *D* are 4.33 and 2.58 × 10^11^ cm·Hz^1/2^·W^−1^ (Jones), respectively, which are acceptable.

**Figure 4:**
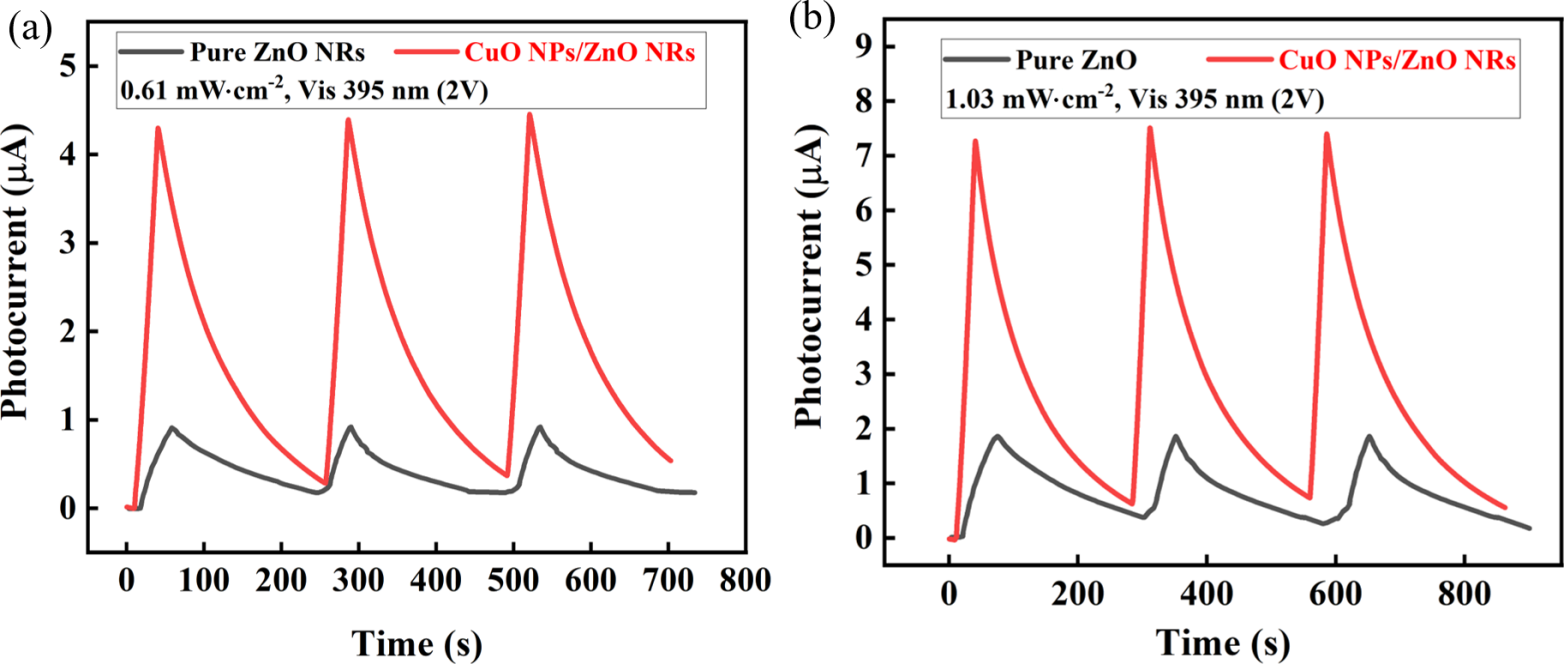
*I*–*t* curves after three cycles under 395 nm visible-light illumination with two power densities: (a) 0.61 mW·cm^−2^ and (b) 1.03 mW·cm^−2^.

The durability of the photodetector based on CuO NPs/ZnO NRs was examined by periodically exposing the device to visible light at 0.61 and 1.03 mW·cm^−2^ with 30 s per cycle (Figure 4). Both experiments show that the photocurrent of the device in both ON and OFF states does not change significantly over the whole cycles. These results indicate the excellent stability of the photodetector during long-term operation. There is a significant difference in stability as well as performance between devices with and without CuO NPs. Decoration of CuO NPs can narrow the bandgap of the heterostructure so that incident photons can quickly generate charge carriers.

**Figure 5:**
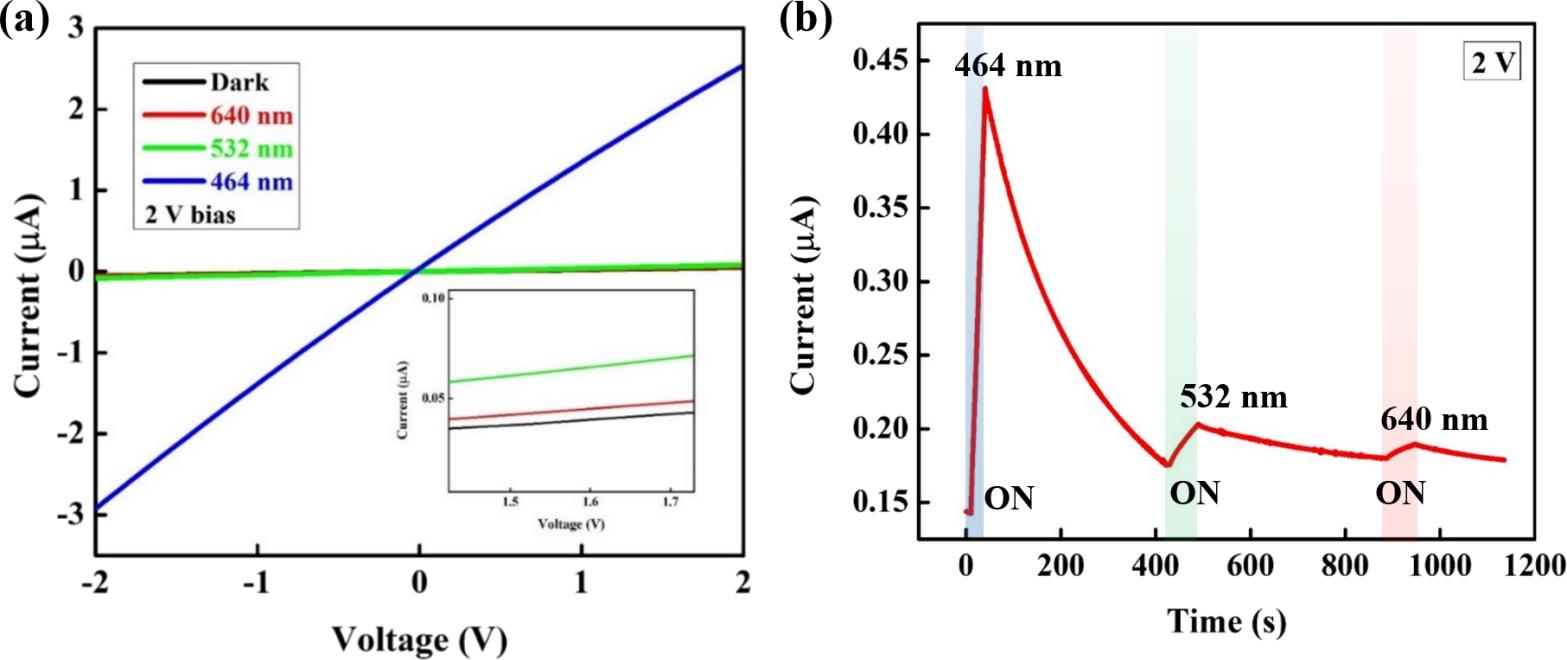
(a) *I*–*V* characteristics and (b) *I*–*t* curves of CuO NPs decorated ZnO NRs photodetector under the illumination of different wavelengths in the visible region.

Interestingly, the CuO NPs/ ZnO NRs photodetector shows also sensitivity to other wavelengths including 464 nm (blue), 532 nm (green), and 640 nm (red). Figure 5a shows the room temperature *I*–*V* characteristics of the CuO NPs/ZnO NRs photodetector under different wavelengths. The photoresponse to wavelengths longer than 395 nm is in accordance with the UV–vis absorbance spectrum discussed above. Figure 5b illustrates the photoresponse of photodetector under 464 nm (130 mW·cm^−2^), 532 nm (149.3 mW·cm^−2^), and 640 nm (133 mW·cm^−2^) illumination as a function of the time. The measured current values are 0.43, 0.2, and 0.18 μA for 464, 532, and 640 nm, respectively.

**Figure 6:**
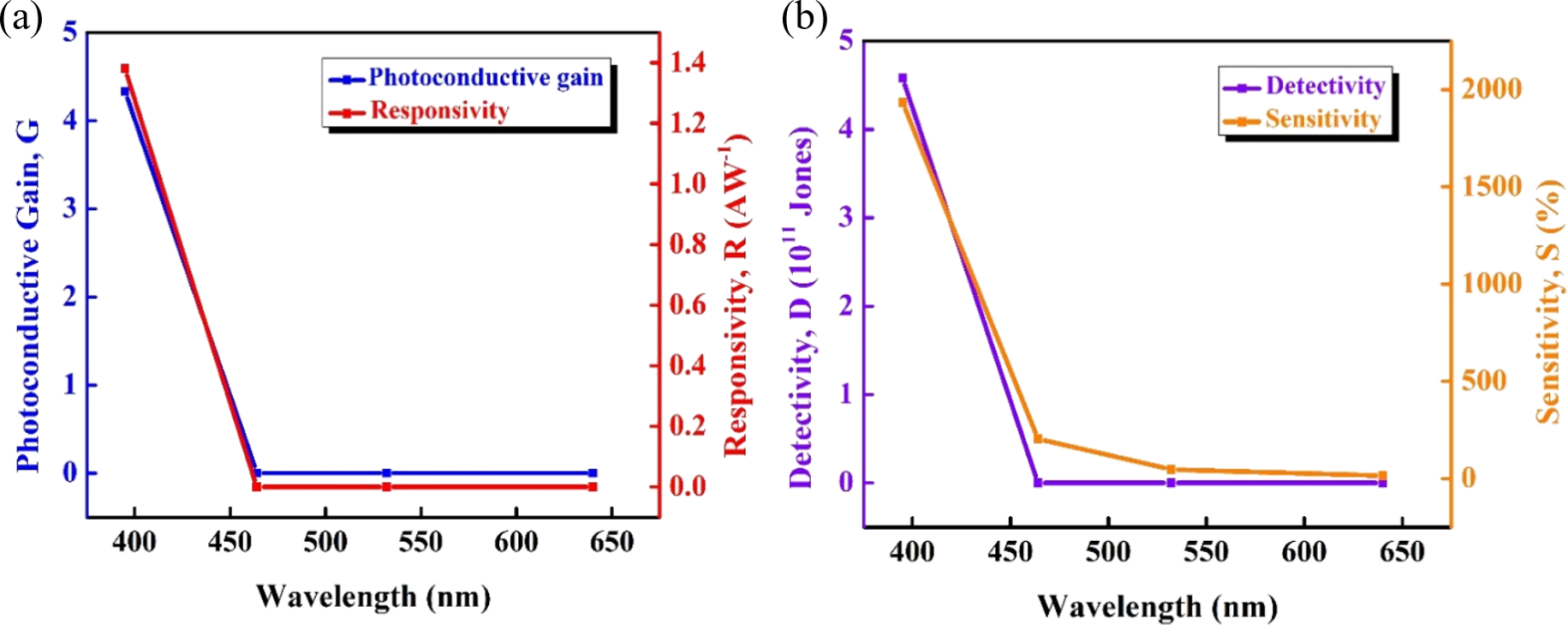
(a) Photoconductive gain and responsivity and (b) detectivity and sensitivity of the photodetector based on CuO NPs/ZnO NRs as functions of the wavelength.

These results indicate that the highest photoresponse value can be achieved when the device is illuminated by blue light. Under green and red light, the photodetector shows low sensitivity. The sensitivity of a device is defined in Equation 4 [51]:


[4] $$S=\frac{I_{\text{ph}}}{I_{\text{dark}}}\times 100\%,$$

in which *I*_ph_ and *I*_dark_ are photocurrent and dark current, respectively. The calculated parameters of the CuO NPs/ZnO NRs photodetector under different light wavelengths, which demonstrate the device performance, are plotted in Figure 6.

We found that the values of *G*, *R*, *D*, and *S* decrease when the wavelength increases. The device is most sensitive to 395 nm, exhibiting weaker signals at 464 nm irradiation and the lowest photoresponse to green and red wavelengths. When illuminating with 464 nm blue light, the recorded responsivity is 0.37 mA·W^−1^; photoconductive gain and detectivity are 9.89 × 10^−4^ and 13.4 × 10^6^ Jones, respectively (Figure 6a). The sensitivity was found to be 1934.5%, 203.4%, 46.6%, and 14.5% at 395, 464, 532, and 640 nm, respectively. Responsivity values of 0.03 and 0.01 mA·W^−1^ are obtained for 532 and 640 nm, respectively (Figure 6b). Since detectivity and photoconductive gain depend on the responsivity [52], the values of *G* and *D* are 0.69 × 10^−4^ and 17.1 × 10^6^ Jones for λ = 532 nm, and 0.19 × 10^−4^ and 5.34 × 10^6^ Jones for λ = 640 nm, which corresponds to the relatively low responsivity values at these wavelengths. Thereby, it can be concluded that the operation range of the CuO NPs/ZnO NRs photodetector could be extended to the visible region.

It can be seen in Table 1 that previous papers about CuO/ZnO-based photodetectors mainly investigated the response in the UV light range and did not demonstrate the sensitivity of the devices to visible light. In this work, we show the performance of our device under exposure to visible light and the operation at lower applied voltages. However, our study’s essential parameters, including *R*, *G*, and *D*, are low compared to previous publications but higher than those that showed photoresponse under visible light. Besides, our photodetector is more effective, easier to synthesize, and less toxic, extending its applicability in everyday life.

**Table 1:** The performance comparison with other ZnO-based photodetectors.

Device	λ (nm)	*R* (A·W^−1^)	*G*	*D* (Jones)	Voltage (V)	Ref.

CuO NPs/ZnO NRs/ITO	365	8.4 × 10^4^	3 × 10^5^	—	5	[53]
p-CuO/n-ZnO NWs	350	0.123	—	—	2	[54]
n-ZnO NRs/p-Si	500	360 × 10^−3^	—	—	4	[55]
ZnO NRs/CuO nanofilm	405	1.24 × 10^−6^	—	9.77 × 10^6^	—	[56]
CuO/ZnO NWs/Ag	564	0.27	—	3.3 × 10^10^	2	[57]
ZnO/ZnS NRs	420	0.49	—	18.3 × 10^12^	4	[58]
ZnO NWs/PbS QDs	500	0.0072	—	4.9 × 10^7^	10	[59]
ZnO/PbS QDs/ZnS	562	0.019	—	—	5	[60]
CuO NPs/ZnO NRs	395	1.38	4.33	4.58 × 10^11^	2	this work
	464	0.37 × 10^−3^	9.89 × 10^−4^	134 × 10^6^		
	532	0.03 × 10^−3^	0.69 × 10^−4^	17.1 × 10^6^		
	640	0.01 × 10^−3^	0.19 × 10^−4^	5.34 × 10^6^		

### Proposed photodetection mechanism

**Figure 7:**
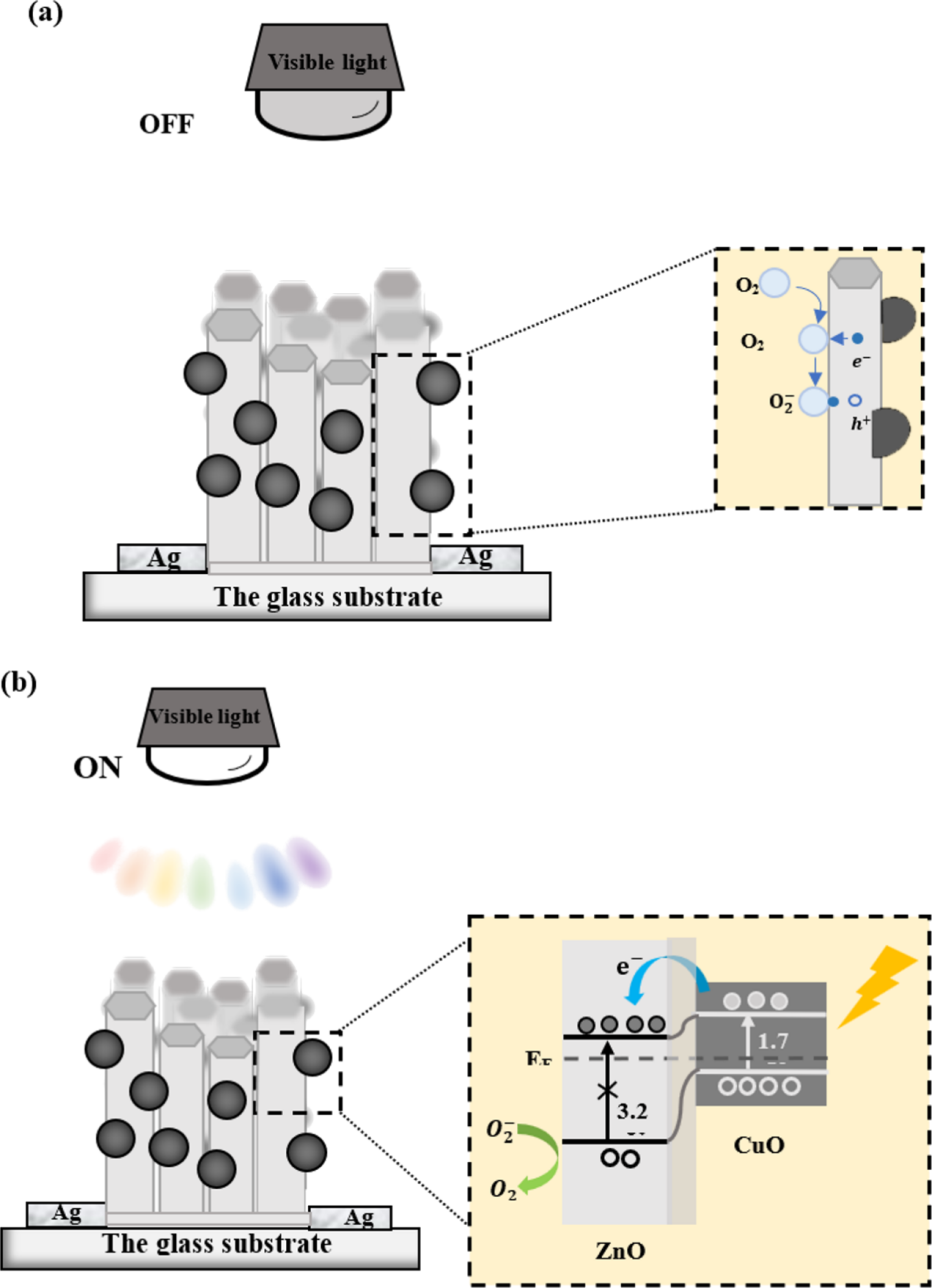
The scheme shows the mechanism for generating the photodetector current in CuO NPs/ZnO NRs under (a) dark conditions and (b) visible-light exposure.

Under dark conditions (Figure 7a), highly electronegative oxygen molecules from the surrounding atmosphere adsorb on the surface of ZnO NRs. These molecules capture the free electrons from ZnO NRs and become negatively charged 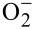 ions [43,61], following Equation 5:


[5] $${\text{O}}_{2(\text{gas})}+{\text{e}}^{-}\to {\text{O}}_{2}^{-}.$$

In addition, decorating CuO NPs onto ZnO NRs forms p–n junctions between the two materials. The heterojunction formation leads to a concentration gradient of charge carriers at the interfaces. CuO NPs act as a p-type semiconductor, in which holes are major carriers. ZnO is a commonly known n-type material, where electrons are dominant. The Fermi levels of both materials will be aligned, which will result in energy band bending at the interface. To achieve the equilibrium state, electrons from ZnO NRs are diffused to CuO NPs while holes are transferred from CuO NPs to ZnO NRs. As a result, a space charge region is created at the heterojunction interface, and an internal electric field is formed.

Under visible-light illumination (Figure 7b), while charge carriers are not generated inside ZnO NRs because of the large bandgap, electron–hole pair generation can occur easily inside CuO NPs as this material has a suitable value of *E*_g_ (ca. 1.7 eV):


[6] $$\text{CuO}+h\nu \to {\text{h}}_{\text{VB}}^{+}\left(\text{CuO}\right)+{\text{e}}_{\text{CB}}^{-}\left(\text{CuO}\right).$$

Since the CB of CuO NPs is higher (more negative) than that of ZnO NRs, photoexcited electrons in CuO easily transfer into ZnO NRs, while holes stay in the VB of CuO NPs:


[7] $${\text{e}}_{\text{CB}}^{-}(\text{CuO})\to {\text{e}}_{\text{CB}}^{-}(\text{ZnO}).$$

Besides the internal electric field, a potential barrier forms because the CB and VB levels of CuO NPs (−4.96 and −3.26 eV vs absolute vacuum scale (AVS), respectively), are higher than those of ZnO NRs (−4.19 and −0.99 eV vs AVS) [62]. It is vital to prevent the recombination of the electron–hole pairs. The electrons are gathered by the Ag electrodes and generate the photocurrent, while holes accumulate near the surface of ZnO NRs to desorb ionized oxygen 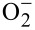 and release gaseous oxygen:


[8] $${\text{O}}_{2\text{ (surface)}}^{-}+{\text{h}}^{+}\to {\text{O}}_{2\text{ (gas)}}.$$

## Conclusion

In this report, a visible-light photodetector based on CuO NPs/ZnO NRs was fabricated by simple and time-saving methods. Spherical CuO NPs were successfully decorated onto ZnO NRs; this enhanced the absorption ability from UV to the visible-light region because of the narrow optical bandgap of the CuO NPs/ZnO NRs heterojunction. The recorded highest photocurrent was 10 μA under 1.28 mW·cm^−2^ illumination at 395 nm and 2 V bias. The maximum values of *R*, *G*, and *D* were 1.38 A·W^−1^, 4.33, and 2.58 × 10^11^ Jones, respectively. The recovery time was 84.64 s, while the response time was about 21.38 s to achieve 63% of the maximum photocurrent value. Simultaneously, the CuO NPs/ZnO NRs photodetector shows photoresponse to other visible wavelengths (464, 532, and 640 nm), and an excellent sensitivity value of 203.4% for blue-light (464 nm) exposure. Therefore, the photodetector based on CuO NPs/ZnO NRs can enable research into broadband optoelectronic devices with simple and low-cost fabrication techniques. The structure can be extensively studied and optimized to achieve higher performance and to reduce response and decay times in the future.

## Experimental

### Materials

The chemical materials used in this report were zinc oxide nanoparticles (ZnO NPs, 99%, Sigma-Aldrich Chemistry), hexamethylenetetramine (HTMA, 99%, C_6_H_12_N_4_, Xilong Scientific), zinc nitrate hexahydrate (Zn(NO_3_)_2_·6H_2_O, 99%, Xilong Scientific), copper(II) nitrate pentahydrate (Cu(NO_3_)_2_·5H_2_O, 99%, Xilong Scientific), sodium hydroxide (NaOH, 99%, Sigma-Aldrich Chemistry), ethanol (C_2_H_5_OH, 99.5%, Chemsol), and acetone (CH_3_OCH_3_, 99.7%, Chemsol).

### Synthesis process

The synthesis process of the ZnO NRs was presented in a previous paper [63]. At the beginning, ZnO NPs (5% dispersion in ethanol) were spin-coated onto cleaned glass substrates at 3000 rpm for 30 s. Then, the sample was heat-treated at 90 °C. ZnO NRs were grown by a hydrothermal method from Zn(NO_3_)_2_·6H_2_O and HMTA (1:1) solution in an oven at 95 °C within 3 h (as presented in Supporting Information File 1, Figure S1). CuO NPs were synthesized via a simple and low-cost sol–gel method. At first, a mixture of 1.21 g Cu(NO_3_)_2_·5H_2_O and 0.8 g NaOH was dissolved in 50 mL deionized water under constant stirring at room temperature. Then, 1 mL HNO_3_ solution was added dropwise into this mixture under vigorous magnetic stirring. After annealing at 180 °C for 18 h, the black precipitate was washed several times and centrifuged (Supporting Information File 1, Figure S2). The formation of nanoparticles was observed through the color change of the solution from blue to bluish-green and finally to black. The sample was dried, and the obtained black powder was CuO NPs. Spray coating was carried out to decorate CuO NPs onto ZnO NRs followed by heat treatment at 90 °C for 1 h to remove the remaining solvent and increase the bonding between nanoparticles and nanorods. To fabricate the photodetector, CuO NPs/ZnO NRs were deposited on a glass substrate initially. Then, silver electrodes with a thickness of 100 nm were directly patterned on the glass substrate by a sputtering process using a shadow mask with 0.3 cm channel length and 0.02 cm channel width.

### Characterizations

The crystal structure of the materials was investigated by X-ray diffractometry on a Bruker D8 Advance diffractometer with Cu Kα radiation (λ = 1.5406 Å). An energy-dispersive X-ray spectrometer (FEI iQUANTA FEG-200) was used to determine the chemical composition of the materials. The morphology of pure CuO NPs and CuO NPs/ZnO NRs was examined by field-emission scanning electron microscopy. UV–vis absorption spectroscopy showed the optical properties of the materials. The photodetector performance was studied through the current–voltage (*I*–*V*) characteristics and current–time (*I*–*t*) curves (Keithley 2400). Visible detection measurements were performed using LEDs with different wavelengths (395, 464, 532, and 640 nm).

## Supporting Information

File 1 Additional figures.
