# Supplementary material for: A visible-light photodetector based on heterojunctions between CuO nanoparticles and ZnO nanorods
Source: Beilstein J Nanotechnol. 2023 Oct 13;14:1018–27. doi: 10.3762/bjnano.14.84 (PMC10616698; doi:10.3762/bjnano.14.84)
Supplement: File 1 — Additional figures. [file Beilstein_J_Nanotechnol-14-1018-s001.pdf]

BEILSTEIN JOURNAL OF NANOTECHNOLOGY

# Supporting Information

for

# A visible-light photodetector based on heterojunctions between CuO nanoparticles and ZnO nanorods

Doan Nhat Giang, Nhat Minh Nguyen, Duc Anh Ngo, Thanh Trang Tran, Le Thai Duy, Cong Khanh Tran, Thi Thanh Van Tran, Phan Phuong Ha La and Vinh Quang Dang

*Beilstein J. Nanotechnol.* **2023,** *14,* 1018–1027. doi:10.3762/bjnano.14.84

## Additional figures

License and Terms: This is a supporting information file under the terms of the Creative Commons Attribution License (https://creativecommons.org/licenses/by/4.0). Please note that the reuse, redistribution and reproduction in particular requires that the author(s) and source are credited and that individual graphics may be subject to special legal provisions.
The license is subject to the *Beilstein Journal of Nanotechnology* terms and conditions: (https://www.beilstein-journals.org/bjnano/terms)

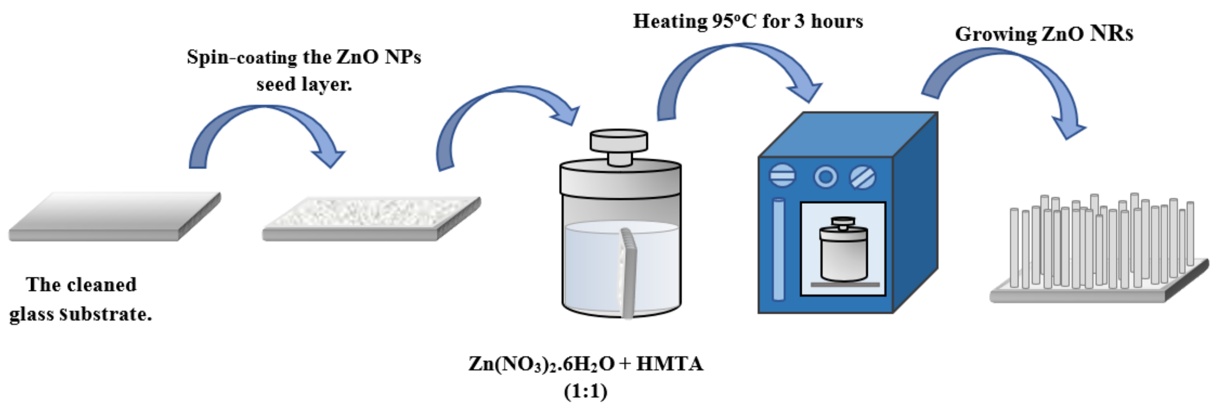


**Figure S1:** Synthesis of ZnO NRs.

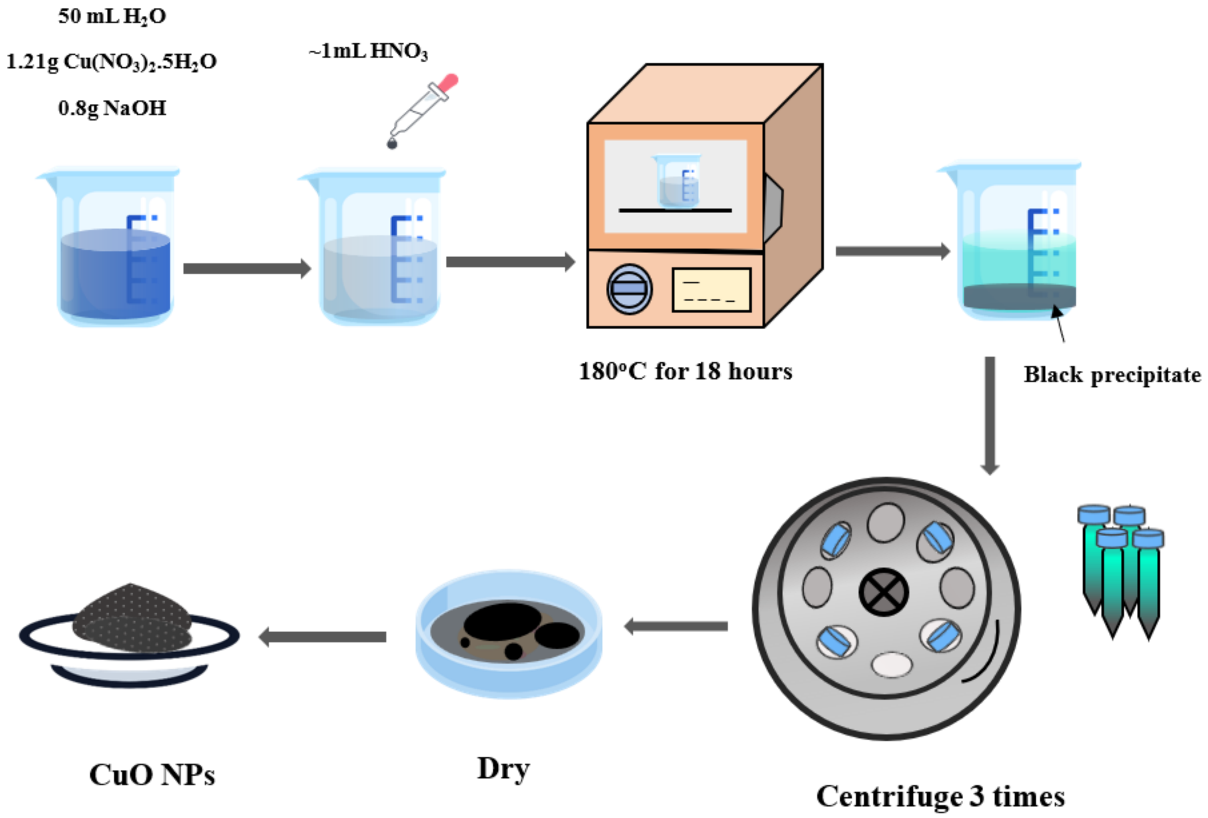


**Figure S2:** Synthesis of CuO NP powder.
